# Comparing Farm Animal Transport Welfare Legislation in Brazil and Germany/EU

**DOI:** 10.3390/ani16142264

**Published:** 2026-07-22

**Authors:** Stefan Timm, Paulo César Maiorka, Alexander Welker Biondo, Louise Bach Kmetiuk, Cristiane Schilbach Pizzutto, Joerg Hartung

**Affiliations:** 1 Brazilian Association for Animal Welfare, São Paulo 04719-030, Brazil; stefan_timm@hotmail.com (S.T.); maiorka@usp.br (P.C.M.); 2 Veterinary School, University of São Paulo, São Paulo 05508-220, Brazil; 3 Department of Veterinary Medicine, Federal University of Paraná, Curitiba 80060-000, Brazil; louisebachk@gmail.com; 4 Laboratory of Ecology and Evolution, Butantan Institute, São Paulo 05503-900, Brazil; cspizzutto@yahoo.com.br; 5 Institute for Animal Hygiene, Animal Welfare and Farm Animal Behaviour, University of Veterinary Medicine Hannover, Foundation, 30559 Hannover, Germany

**Keywords:** animal welfare, livestock road transport, legislation, Brazil, Germany, European Union, EU–Mercosur

## Abstract

Animal transport connects farms with slaughterhouses, breeding units and production sites, but remains one of the most debated animal welfare issues of recent decades. These debates also touch on the EU–Mercosur agreement in the areas of agribusiness and animal farming. The aim of this article is to examine the legislation of Brazil and Germany and the European Union (EU) regarding animal transport, using specific examples to identify differences and similarities that may form the basis for joint discussions on this area of animal welfare. The EU Transport Regulation for the Protection of Farm Animals applies in all EU member states (Council Regulation (EC) No 1/2005). In Germany, the Animal Welfare Transport Ordinance transposes EU law into national law. The transport times depend on vehicle type and equipment and vary according to the animal species to be transported. The basic maximum travel time is 8 h for commercial transport and distances longer than 65 km. In Brazil, there exists a wide range of laws, regulations, decrees and guidelines governing the protection of animals during transport in the different Brazilian states, mostly coordinated by the Brazilian Ministry of Agriculture and Livestock (MAPA). Allowed transport times in Brazil vary largely from 4 to 12 h between single state regulations and are less regulated in detail. Maximum Transport durations can be repeated several times after appropriate stops for animal feeding and watering. Similarly to Germany/EU, only animals “fit for travel” are allowed to be transported and the responsibility for the transported animals ends once the last animal has been unloaded at its place of destination. Temperatures in Germany/EU are generally moderate, whereas Brazil and South America have tropical and subtropical climates. Furthermore, the distances between farms and abattoirs in Brazil are generally greater, and transport vehicles often have to travel on unpaved (gravel) roads, e.g., to reach the farms. The key to animal-friendly transport lies in loading healthy, “transport-fit” animals on suitable vehicles with sufficient space and necessary equipment, and licensed and committed drivers. This has to be supported by appropriate legal provisions. Animal welfare laws in both Brazil and Germany/EU aim to ensure the best possible welfare of animals during transit under the given circumstances of transport, in order to prevent pain, suffering and injury. This shared commitment to animal welfare provides a substantiated basis for mutual understanding and cooperation in livestock farming, which may prove helpful in the discussions on the agricultural chapter of the EU–Mercosur Agreement.

## Simple Summary

The article compares animal welfare transport regulations in Brazil and Germany/EU to understand their similarities and differences. In the EU, transport is strictly regulated with clear rules on travel times, breaks, and driver training. In Brazil, rules exist but vary between states and are generally less detailed. Transport conditions also differ due to geography and climate: Brazil often has longer distances, warmer climates, and rougher roads, while Europe has shorter routes and in general moderate climatic conditions. Despite these differences, farm animal producers in both regions share the stated objective of protecting the animals during transport—ensuring animals are fit for travel and minimizing injury, pain and suffering during transit. Overall, the paper suggests that this shared commitment to animal welfare is a substantiated basis for mutual understanding and cooperation in animal farming, which may prove helpful in the discussions on the agricultural chapter of the EU–Mercosur Agreement.

## 1. Background

The trade agreement between the European Union (EU) and the Mercosur countries (Argentina, Brazil, Paraguay and Uruguay) was approved for provisional application in February 2026 (Official Journal of the European Union, OJ L 185, 27 February 2026). The agreement is set to enter into force provisionally on 1 May 2026 (Official Journal of the European Union (OJ L, 2026/868), despite numerous protests, particularly from farmers’ organizations in several EU Member States. One of the points of criticism is the concern that animal welfare standards for the rearing, transport and slaughter of animals in the South American partner countries do not meet those of the EU. It therefore seemed sensible to examine the animal welfare regulations of the contracting parties in somewhat greater detail. For an initial comparison, Brazil and Germany, both having animal welfare as a goal in their constitutions, were selected as major livestock-producing countries with detailed welfare regulations for farm animals, including their transport.

For the purposes of this article, we will focus on the regulations governing the road transport of farm animals, with little mention of other means of transport.

## 2. Introduction

Animal transport connects farms with slaughterhouses, breeding units and production sites, but remains one of the most debated animal welfare issues of recent decades [1,2,3]. This may also be linked to the high number of animals transported. As an example, approximately 6.7 billion chickens, 58 million pigs and 43 million cattle were slaughtered in Brazil [4] and around 697 million chickens, 45 million pigs and 2.8 million cattle in Germany [5]. These data are only estimates because data on the transport of live animals are fragmented at EU level [European Parliamentary Research Service—EPRS 2021] and also in Brazil. The main reason at EU level for this is that EU legislation does not oblige Member States to collect and report data on the transport of live animals. The Transport Regulation merely obliges Member States to report on the checks carried out [6]. Most trade in live animals between the Member States of the EU takes place by road, accounting for 70% in terms of weight and 65% in terms of market value [6]. In Brazil, too, most farm animals is transported by road, although a significant proportion is also transported by river vessels and by sea—mostly for export. However, these animals are also usually transported to the port by road vehicles.

Transport has been known to trigger stress reactions in animals and can significantly impair their health and well-being [3,7]. Additionally, stressful transport can also have a negative impact on the meat quality of the slaughtered animals [8,9,10]. Moreover, animal transport is frequently associated with the loss of animals. In European conditions, dead on arrival (DOA) rates range between 0.02% and 0.03% for transported adult cattle and dairy cows, around 0.06% for pigs, and are usually higher in poultry—between 0.42 and 0.43% [10]. A dead-on-arrival (DOA) rate of approximately 0.08% was reported for pigs transported to slaughterhouses in Brazil [11].

Broom [12] provides an overview of the multitude of factors that can influence animal welfare during transport and describes that welfare can range from good to poor across fluctuating phases. A recent report “Transport of live animals in the EU: challenges and opportunities” of the European Court of Auditors confirms in detail the full complexity of conditions and factors acting on transported animals and that road transport is by far the most dominating means of transport for farm animals (European Court of Auditors 2023) [6].

Inappropriate loading and reckless driving can result in injury, while transporting unfit animals may lead to their death or the spreading of infectious diseases [3,13]. In fact, transport-related mortality has been a key indicator of animal welfare, commonly associated in Europe with seasonal effects and transport distance [14].

**Figure 1:**
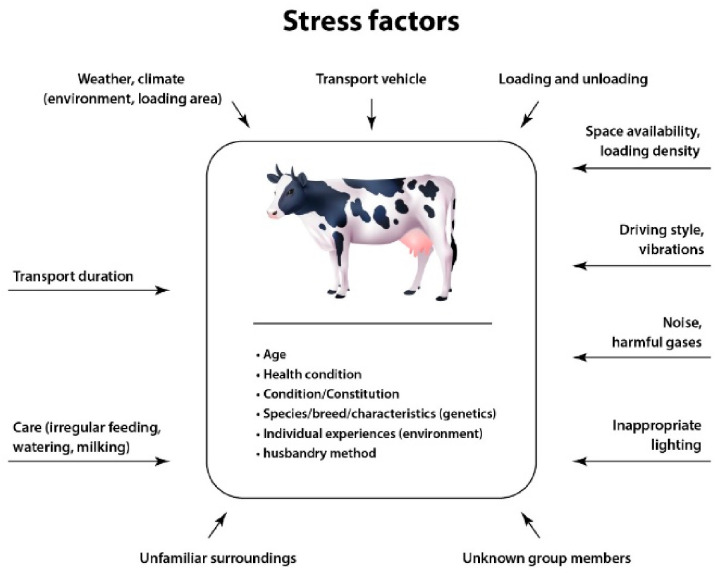
Stress factors during animal transport adapted from Gayer et al., 2016 page 26 [15].

The importance of careful and species-appropriate transport becomes clear when one considers the multitude of factors to which animals may be exposed during such journeys (Figure 1), and which can severely impair their health and well-being.

Animal welfare has a long history in Europe. The British Parliament passed the world’s first animal welfare law in 1822 to protect horses and cattle [16]. The first European Council Directive (77/489/EEC) on the protection of animals during transport was adopted by the European Economic Community in 1977 [17] and overhauled in 1991 [18]. The Council Regulation (EC) No 1/2005 [19], which came into force in 2005, applies in the 27 EU Member States, as well as in Iceland and Norway [20].

The European Food Safety Authority (EFSA) has recently published an animal transport guideline and recommendations, describing and discussing the most important influences and stress factors on animal health and welfare during transport [10]. These guidelines reflect the outcomes of several important EU-funded research projects on animal welfare during transport such as CATRA Minimizing stress inducing factors on cattle during handling and transport to improve animal welfare and meat quality (2000–2003), the ‘Welfare Quality’ project (2004–2009) and the AWIN (Animal Welfare Indicators) project (2011–2015) that all have provided deep insights into the health, behavior and welfare of animals in transit, as well as into vehicle design, transport procedures, handling and conditions. The projects developed protocols for the application of animal-based indicators to assess an animal’s condition at various life stages, including transport, enabling an assessment of welfare during handling and transport, taking into account factors such as pain, injury and stress that are particularly relevant to transport conditions, and considered thermal transport environment. Findings from these research projects, together with practical experience, were incorporated into EU and German legislation.

Likewise, Brazil launched a public hearing in 2025 for a federal ordinance on livestock transport, mostly to ensure animal protection and welfare during animal transportation [21]. A Germany–Brazil partnership has organized international events called “Don’t Forget the Animals”, a forum of specialists focused on contemporary challenges on animal health, animal welfare and sustainability, based on the 2022 UN Resolution on One Health towards animals [22]. The international group established in 2025 the Brazilian Association of Animal Welfare (ABBEA), aiming to assess, qualify and certify good practices on animal welfare and production, in compliance to international and EU animal welfare standards [23].

Furthermore, the World Organization for Animal Health (WOAH) has published animal welfare standards for transport, which underscores the global importance of animal welfare during transport [24]. These transport guidelines focus on minimizing stress, injury, and disease spread, emphasizing shared responsibility among personnel, proper infrastructure, and journey planning. These guidelines also stress the importance of fitness for travel, careful handling and vehicles, that must be safe, secure, easy to clean, and designed to protect animals from injury.

Despite a considerable bulk of knowledge, and an awareness of the importance of animal welfare during transport, there is still a lack of binding international legislation that is observed by all countries worldwide.

## 3. Materials and Methods

For the present study, all available laws, regulations, decrees, resolutions and guidelines for transportation of farm animals in Brazil were searched, reviewed, and compiled in three books, comprising more than 4000 pages (Timm et al. 2021, 2025 [25,26]), because an official centralized register is missing presently in Brazil. For Germany Council Regulation (EC) No 1/2005 [19], the German Animal Welfare Transport Ordinance 2021 [27] and the Federal Animal Welfare Act [28] were regarded.

## 4. Results

### 4.1. Legal Provisions

#### 4.1.1. Germany (And the European Community)

In 2005, the European Union (EU) issued Council Regulation (EC) No 1/2005 of 22 December 2004 on the protection of animals during transport and related operations [19], which regulates animal transport within the EU. Germany implemented this regulation into national law in 2009, through the German Animal Welfare Transport Ordinance (TierSchTrV) [27]. In these regulations, the legal responsibility for the animals belongs to the person currently in possession of them. Animal transportation, and thus also the transport time, begins with the loading of the first animal in a group or the individual animal to be transported, and ends when the last animal sets foot on the ground at destination. During unloading, e.g., at the abattoir, cattle, pigs and other large animals are examined by a vet at the ramp, who also verifies the accompanying documents [29]. Injured and sick animals are separated from the herd and either treated or put down, and do not enter the human food chain.

#### 4.1.2. Brazil

Brazilian regulations for live farm animal transport comprise a large number of laws, decrees and resolutions, mostly issued by the Brazilian Ministry of Agriculture and Livestock [30]. The technical requirements in Brazil for vehicles of farm animal transport or “live animal transport vehicle” were updated by the National Transportation Council on 18 June 2020, by Resolution 791 (formerly CONTRAN 675/2017) [31]. This resolution specifically mentions the Guidelines for issuing Animal Transit Permits (GTA = Guia de Trânsito Animal) by the Ministry of Agriculture and Livestock (MAPA).

The GTA is the most important document for animal transport in Brazil. It is Brazil’s official and mandatory animal transit document, used for sanitary control and traceability. A GTA must accompany almost all live animal movements, whether intrastate (within the same state) or interstate, including aquatic animals [32]. The GTA is available in printed and electronic format (MAPA 09/2021) [33].

Specific requirements for farm animal transport, such as loading ramp inclination, fitness for travel and transport duration are regulated by MAPA 46/2011 (regulation for organic animal and plant production systems) [34]. Regulations for the export of live cattle, buffalo, sheep and goats intended for slaughter or breeding can be found in MAPA 46/2018 [35]. MAPA 113/2020 regulates good animal welfare and management practices on commercial pig farms [36]. However, the instructions are limited to pigs, live ruminants to be exported or farms wishing that their product be traded as organic. Fitness for travel applies to all animals throughout the documents. Specifications on transport duration is regulated mainly at the state level, which can create a problem in jurisdictional enforcement.

Only 14 States and the Federal District (where the capital Brasília is located) have animal protection laws with specific farm animal transport regulations. This further complicates the uniform monitoring of animal welfare. The responsibility for the well-being of the animals during the entire process within the Brazilian territory is defined by Decree 9.013/2017 (regulation for the industrial and sanitary inspection of products of animal origin) [37,38,39]. Slaughterhouses play an important role in this regard. Their mandate for animal welfare applies specifically from the loading point of origin through the time of slaughter. This differs from Germany, where the transporter is responsible for the animals from loading to unloading.

The establishment must adopt measures to prevent animal abuse and implement actions to protect animal welfare, from loading until slaughter. In addition, mandatory actions include notification before arrival, transit documentation control on arrival, sealed transport vehicles (in order to ensure animal origin), and disembarkation and housing in appropriate exclusive facilities, respecting species particularities.

Most transport regulations in Brazil are focused on zoo sanitary control and customs inspections rather than on animal welfare itself: day-old ostrich chick import (MAPA 44/2002) [40], importation of live animals and animal breeding material (MAPA 01/2004) [41], animal health requirements for Mercosur Member States regarding pig importation for breeding (MAPA 63/2013) [42], national transit of swine, their products, by-products and genetic material for southern (MAPA 33/2014) [43] and other states (MAPA 27/2015) [44], International Agricultural Surveillance System (MAPA 39/2017) [45], and animal health requirements for Mercosur Member States regarding the importation of domestic poultry, hatching eggs and day-old chicks (MAPA 62/2018) [46].

### 4.2. Transport Phases

Even though the transport process is usually divided into three different phases—loading, journey (with/without breaks) and unloading [15]—this process initiates during the pre-transport or preparatory phase, sometimes days before loading animals onto the transport vehicle.

#### 4.2.1. Pre-Transport (Preparation)

Pre-transport preparation for farm animals involves critical health checks, biosecurity measures, and welfare considerations to minimize stress and disease risk before transport, including pre-export inspections, appropriate feeding and water, proper handling, quarantine if needed, and having all the required health certificates to ensure a smooth and safe journey [47]. According to these guidelines, key steps have focused on confirming animal health, preparing facilities, and planning logistics to prevent unnecessary stress and contamination.

Some requirements are species-specific, such as 16–24 h pig fasting to slaughter, as part of on-farm preparation prior to transport. This measure aims to prevent travel sickness and potentially even deaths, particularly in hot weather and stress-susceptible pigs. However, pigs are known to become more difficult to handle after 18 h of fasting due to hunger-induced frustration, fatigue, and excitement [48]. Not surprisingly, pig transport management is a major influencing factor in stress and pig welfare, along with direct human–pig interactions and slaughter procedures, as a systematic review has recently shown [49].

However, the Brazilian regulation on animal welfare in pig farms does not mention pre-transport fasting (MAPA 113/2020) [36], which is foreseen in two state laws, (Ceará) 17.729/2021 and (Piauí) 8.364/2024 [50,51]. Fasting and water restriction periods from the point of origin to the final destination fall under the responsibility of the slaughterhouse. Even though the regulation for the industrial and sanitary inspection of products of animal origin (Decree 9.013/2017) [38] mentions fasting, only general recommendations are given, with no details on pre-transport procedures, only prohibiting slaughter without animal rest, fasting and water restrictions, without specifics. There is room here to improve animal welfare regulations in the future.

In the EU, fasting prior to transport is not mandatory for farm animals [19]. However, there is a requirement for pigs to have continuous access to water (and no food) on road vehicles, indicating that pre-transport fasting is a common management practice for pigs.

##### Fitness for Travel

One of the most important prerequisites for transport is that animals are fit for travel, as described in Council Regulation (EC) 1/2005 [19]. In the case of long-distance farm animal transport, the journey must be inspected and cleared by an official veterinarian in advance, including the so-called journey log. The responsible (EU) authorities are notified about the approximate ETA at the destination. During clearance, all veterinary-relevant criteria are checked such as animal species, age, health status, general condition, and freedom from (evident) disease [15].

Fitness for travel requirements in Brazil can be found for organic farms (MAPA 46/2011) [34], live ruminants to be exported (MAPA 46/2018) [35], pigs (MAPA 113/2020) [36], and at a state level. The regulation for animal welfare on pig farms (MAPA 113/2020) [36] prohibits loading pigs showing signs of pain or deemed unfit for transport onto vehicles, such as young animals with an unhealed navel, sows in the final third of gestation or up to ten days postpartum, pigs submitted to surgical procedures within ten days prior to transport, and pigs with emaciated, fractured or dislocated limbs, that are unable to stand or move.

These criteria correspond roughly to the rules set out in the Council Regulation 1/2005: Animals are strictly considered unfit for travel, if they are

Unable to walk independently;
Unable bear weight evenly on all legs;
Unable to move without pain;
Suffer from severe injuries;
Have large open wounds;
Have heavy bleeding;
Have significant organ or uterine prolapses;
Are in an advanced stage of pregnancy (typically beyond 90% of gestation);
Are likely to give birth during the trip;
Have given birth in the preceding 48 h;
Suffer from a systemic illness;
Have central nervous system symptoms;
Have high fever or are very young (e.g., calves under 10 days old, lambs under 1 week, or pigs under 3 weeks) and being transported over long distances.

An animal with a slight illness or injury may be transported if the condition is minor, they can walk, the transport will not cause extra suffering, or the transport of a sick animal is ordered by a veterinarian. A casualty animal might be transported directly to the nearest slaughterhouse for emergency slaughter, but this usually requires the owner to complete a specific declaration detailing the illness or injury.

The regulation for the export of live cattle, buffalo, sheep and goats intended for slaughter or breeding (MAPA 46/2018) [35] only instructs that the road transport of animals respect their health status, general well-being, physical fitness for travel and their ability to cope with transport stress, including very young, old, gestating and lactating animals. This is a restriction (rather than prohibition), much like the regulation for organic animal and plant production systems (MAPA 46/2011) [34], which establishes that animal transport, pre-slaughter, and slaughter (including sick or discarded animals) must respect specific legislation, principles of animal welfare, reduction in painful processes, and humane slaughter procedures. In practice, this means that there is a restriction but not a ban, which leaves some room for compliance.

In total, 19 (70.1%) out of 27 Brazilian territories have laws addressing animal transport, of which only 14 explicitly prohibit the transport of animals considered weak, sick, injured, or in advanced stages of gestation, except for veterinary care or in emergencies. Here, too, there is room for improvement regarding animal welfare during transport.

##### Documentation

In Germany, once animals are fit for transport, the transporter must have all the required documents in place before loading [15] for a long transport: Type II transport operator license from the competent authority (veterinary office), certificate of competence for drivers and attendants, registration certificate for the means of transport, logbook (journey log), species-specific documents (e.g., cattle passports), and disinfection control book.

In contrast, Brazilian law only requires three documents: truck driver’s license (class D), proof of registration of the live animal transport vehicle (VTAV) and Animal Transit Permit (GTA).

##### Transport Vehicle Specifications (Means of Transport)

The equipment of the vehicles is important for animal-friendly transport. A comparison of regulations in Table 1 regarding animal transport vehicles in both countries shows similarities and some comparable instructions, such as how transport-like vehicles should be designed, constructed, maintained and operated.

In Germany (and other EU countries), provisions are required to ensure regulated animal access to feed and water, along with a comfortable microclimate inside the vehicle, comprising adequate temperature, humidity, and ventilation conditions. An early EFSA Opinion 2004 gives temperature limits inside animal transport vehicles [52] which were amended and specified by several reports on animal welfare of cattle, small ruminants and pigs during transport in 2022 [53,54,55]. Generally, under European Union Regulation EC 01/2005 (https://www.animals-angels.de/en/neuigkeiten/titel/2020/animal-transports-during-heat.html, accessed on 6 July 2026), the optimal microclimate inside the vehicle should range between 5 °C and 30 °C with a +/−5 °C tolerance for the entire duration of the trip. In times of increasing ambient temperatures worldwide and increasing heat waves, more ventilation capacity, cooling systems and prudent travel logistics are required to avoid heat stress. Thus, for particularly sensitive animals like horses, special equipment exists for transport vehicles to be equipped to control the climatic conditions and other parameters in the cargo hold [56]. Poultry and other animals like rabbits can also suffer significantly from heat stress during transport [57]. Such thermal impact on animals in transit should be regarded worldwide as an important welfare risk for transported animals. The thermal stress experienced by animals during transport across a wide range of environments is recognized globally as a significant animal welfare risk. Useful guidelines for better protecting animals against transport-related heat stress are found in the international animal welfare standards of the WOAH [24]; these provide practical advice on ventilation and microclimate monitoring, recommend transport protocols, and thereby help mitigate heat-related risks. These are also used in Brazil.

Regulations in Germany/EU have a clear quantitative characteristic, while Brazilian requirements are more qualitative, such as the air circulation inside the vehicle that must guarantee “necessary ventilation” for animal welfare (CONTRAN 791/2020) [31]. Regulations on animal transport must be objective, without subjective terms such as adequate space and proper handling, which may be interpreted differently by different individuals [58]. Therefore, the enforcement of the regulations is of crucial importance for the well-being of the animals.

### 4.3. Loading and Unloading

Animal loading and unloading represents a significant injury risk, considering the changes in heart rate and cortisol release in animals [59]. Thus, loading and unloading should be carried out in a particularly calm and non-stressful manner. Suitable loading ramps with a gradient no greater than 20° are also helpful [15]. However, CONTRAN 791/2020 only requires a species-appropriate loading gate (which includes the ramp), without describing or specifying its characteristics [31]. The regulation of animal welfare on pig farms (MAPA 113/2020) [36] is the only one to establish a maximum ramp inclination angle of 25°. It also requires that animals be handled by trained personnel during loading and unloading. The regulation for exporting live ruminants (MAPA 46/2018) [35] requires exit points to have a workforce trained in animal welfare protocols as described in the Terrestrial Animal Health Code of the World Organization for Animal Health (WOAH) [24].

In Germany, Council Regulation (EC) 1/2005 stipulates a maximum ramp angle of 20° (36.4%) for pigs, calves and horses and 26° 34′ (50%) for sheep and cattle [19]. If the slope is steeper than 10° (17.6%), ramps must have an auxiliary system, such as foot battens, ensuring that animals can climb up and down without risk or difficulty. In addition, this regulation requires drivers and handlers to have successfully completed training and passed an examination approved by the competent authority, ensuring an independent and unbiased evaluation. Training courses include the technical and administrative aspects of legislation concerning animal protection during transport, animal physiology, hydration and dietary needs, animal behavior, animal handling, driving protocol, emergency care, and safety considerations for personnel. Not surprisingly, adequately trained personnel are considered to be a major contributor to the reduction in stress during transport, with direct impact in meat quality [60].

### 4.4. Journey

The major point of concern during the road transport of farm animals both in Brazil and Germany is the prevention of health impairment caused by loading animals not really fit for travel, improper handling during loading, too high animal density in the vehicle, rough driving, particularly on poor roads, and poor ventilation causing heat stress. From a legal perspective, Germany is subject to a relatively detailed and binding framework under Council Regulation (EC) No. 1/2005, implemented nationally through German animal transport law. This regime requires animals to be transported in a way that is unlikely to cause injury or unnecessary suffering and, for long journeys, type II vehicles to provide bedding, water and feed access, adequate ventilation, temperature monitoring, and protection against extreme weather. Climatic conditions must be considered already at the planning stage, including journey timing, stocking density, ventilation, and contingency measures. For long-distance transport, EU law requires the vehicle microclimate to remain within a temperature range of approximately 5 °C to 30 °C, with a limited tolerance of +/− 5 °C, which makes heat-period transport legally problematic unless the vehicle is technically capable of maintaining acceptable conditions.

Brazil, by contrast, has a less specific and less harmonized federal legal framework for animal welfare during terrestrial transport. The Brazilian Ministry of Agriculture recognizes Normative Instruction No. 56/2008 as establishing good-practice recommendations for farm animal welfare in production and transport systems, but these rules are framed more as welfare guidance than as a detailed temperature-based transport regime comparable to EU law. Comparative analysis for the European Parliament has also noted that, in Brazil, animal movement documentation is required, including information on destination, animal health, and transport purpose, but specific animal welfare provisions and penalties for transport are limited at federal level, with enforcement varying between states and municipalities. For live animal exports, Brazil has adopted more specific requirements, including references to international animal welfare standards, but these do not amount to a general nationwide system equivalent to the EU/German rules for all domestic road transport.

Accordingly, in both countries, it is recommended that drivers drive carefully and take advantage of good roads and milder climatic periods, such as cooler hours of the day or cooler months of the year, while adapting stocking density and vehicle microclimate to external weather conditions. During high summer, transport to slaughterhouses should preferably take place at night or in the early morning; otherwise, loading density should be substantially reduced and ventilation actively managed. During low winter, excessive cooling should be avoided, ventilation should be adjusted, and maximum permissible loading densities should not be exceeded. Practical measures include parking loaded vehicles in the shade, avoiding prolonged stationary periods in direct sunlight, and, where breaks are unavoidable, unloading animals into suitable areas where they can actively seek protection from solar radiation.

In general, Germany is regarded as the country with the stricter, more binding legal requirements, whilst Brazil has a system in which, although nationwide guidelines exist, detailed, enforceable animal welfare standards in the transport sector are less uniform.

#### 4.4.1. Space Allowances

The amount of space provided to animals critically impacts animal health and welfare [61]. Allometric principles and equations are used to estimate the static space requirement for animals to feed, drink, stand up and lie-down, creating the basis for establishing animal space requirements during transport [61].

In Brazil, animal density should comply with the MAPA space allowance specifications (CONTRAN 791/2020) [31], which also contains regulations on vehicle construction, waste containment and ventilation [31]. For ordinary domestic cattle transport, MAPA’s Good Management Practices: Transport (Boas Práticas de Manejo: Transporte) is framed as guidance rather than a detailed enforceable density regulation. It recommends calculating the number of cattle by compartment using the linear space per animal and the average live weight [62]. MAPA published, in 2025, a public-consultation draft for animal welfare rules during transport with a general density formula for road, rail and maritime transport: S = k × P^(2/3), where S is surface per animal, P is live weight, and k varies by species (Portaria SDA/MAPA nº 1.280/2025, Portaria SDA/MAPA nº 1.295/2025).

The clearest binding MAPA density rule is in Instrução Normativa MAPA nº 46/2018 [35], mainly for live animal exports. It requires road transport to use vehicles appropriate to the species and to respect animal welfare principles and the loading densities recommended in Annex 01 and is intended to meet importing country regulations. The same density reference is also used for maritime/fluvial and air export transport.

Table 2 compares space allowances for cattle when transported by land in Germany and Brazil. A fairly high degree of agreement can be observed among the countries. The requirements are smaller in Brazil for cattle weighing up to 200 kg (MAPA 46/2018) [35], while they are approximately the same for heavier animals. The space allocated for the animals must ensure that they all have sufficient space and do not fall onto one another during unavoidable braking maneuvers. The figures for Brazil are maximum values, because heat stress is of concern when the lorry is loaded with too many animals. Lower densities are not seen as a problem.

#### 4.4.2. Duration

In Germany, the permitted duration of animal transport is one of the most debated sections in the German Animal Welfare Transport Ordinance (TierSchTrV) [27], which differs significantly to Brazilian regulations. All animal transport in Germany has to be carried out in suitable vehicles. The TierSchTrV [27] and the overarching Council Regulation (EC) No 1/2005 [19] apply to commercial transport of farm animals.

The requirements for vehicles and drivers vary depending on the distance traveled. If the transport is part of a commercial activity, e.g., by a trader or farmer, and covers a distance greater than 65 km, the driver must hold a certificate of competence, and the vehicle must be registered with the relevant authority. Transport using an approved standard vehicle (vehicle type I) must end after 8 h, with the animals being unloaded for at least 24 h. If animals need to be transported for longer than 8 h (long transport), special vehicles (vehicle type II) with forced air circulation and the ability to provide animals with feed and water must be used. Long transport duration depends entirely on the species being transported.

Using cattle as an example, long transport in Germany allows for an initial journey of 14 h, followed by a break of at least 1 h for feeding and watering. The journey can then continue for another 14 h. After these 29 h, the animals must reach their destination or be unloaded at an approved feeding station and rest there for at least 24 h with food and water. After that, the journey may continue at the previously described pacing.

The transport journeys are additionally interrupted by the drivers’ breaks. The driver must take breaks of at least 45 min approximately every 4 to 4.5 h in accordance with EU Social Regulation for Professional Drivers (Regulation (EC) No 561/2006), with total driving time limited to a maximum of 9 h per day. During breaks, drivers are required to check on the animals. When Type II vehicles are staffed by two drivers for long-distance transport, they can drive in alternating shifts. In exceptional cases, such as unforeseen traffic jams or extremely bad weather, driving time may be exceeded without the risk of a penalty.

In Brazil, maximum transport time for the animals is defined but difficult to implement consistently, because it differs from state to state significantly. For instance, the regulation on exporting live ruminants (MAPA 46/2018) [35] states that the road transport time of the animals must (in any situation) respect the maximum limit of 12 h between the pre-embarkment facility and the point of exit out of the country (usually a harbor). However, it also states that in exceptional cases and at the discretion of competent authority, a longer time may be authorized. Furthermore, should the pre-embarkment facility not be the point of origin, transport time between the point of origin and pre-embarkment facility would remain unregulated. Future legislative amendments should provide clarity.

Only 14 of 26 Brazilian states and the Federal District have a duration limit for farm animal road transport, but maximum transport time widely varies. Table 3 summarizes the different regulations on travel times and mandatory breaks. Some states prohibit transporting animals for over 4 h, others for up to 12 h without feed and water. It is important to consider that most vehicles in Brazil are not equipped to feed and hydrate animals without stopping. They are required to stop after 4 h in Amazonas, 6 h in São Paulo, Maranhão, Sergipe, Rio Grande do Norte, Ceará, and Piauí, and 12 h in the Federal District of Brasília. For organic farms, there is a nationwide limit of 12 h for animal transport.

Other states prohibit transporting animals for over 6 h or 12 h without proper rest, meaning it is mandatory to stop after 6 h in Rio de Janeiro and after 12 h in Paraná, Santa Catarina, Espírito Santo, Pernambuco, Tocantins, and Rio Grande do Sul. The way these durations are to be applied or offset against each other for domestic interstate transport is legally unclear.

Journey length in Germany/EU is also complex but clearly formulated. The basic maximum travel time is 8 h for commercial transport and distances longer than 65 km. Transport of cattle, as already mentioned above, can be extended to 29 h with breaks (14 h + 1 h break + 14 h). Pigs can be transported for up to 24 h (constant access to water), poultry for 12 h, one-day-old chicks for 24 h (insulated lorries) and spent hens for up to 10 h. Specific regulations exist for the long-distance transport of certain animal species in specialized transport vehicles: 19 h (9 + 1 + 9) for calves > 14 days old; lambs > 1 week old; piglets > 10 kg. Breaks are determined by rules given in the Council and German Regulations, also on feeding and watering, and by the social regulation for professional drivers (Regulation (EC) No 561/2006) requiring 45 min breaks after 4.5 h of driving. The given transport times can be repeated with the same animals only after they are unloaded for 24, accommodated in appropriate housing and fed and watered.

## 5. Discussion

This article focuses on statutory animal welfare regulations in Brazil and Germany/EU governing the transport of animals by road, which is the most common form of animal transport [76,77]. This brief comparison of the farm animal transport welfare legislation reveals differences, as well as similarities and commonalities between Brazil and Germany/EU. The first notable difference between both countries is the volume of legislation regarding farm animal transport. Germany has very straightforward central regulations (only two), while Brazil has a large number of legal provisions sparsely spread between federal and state jurisdiction, being often limited to a specific animal category or activity. This presents a significant challenge in enforcement, for it is confusing and leaves much open to interpretation.

Animal transport duration in Brazil generally varies between 4 h and 12 h, while in Germany such duration is based on vehicle type and travel times, along with transported animal species such as cattle, pigs or poultry. Another important difference concerns animal transport breaks, which has no current precise regulation in Brazil, allowing maximum transport time to be repeated several times after appropriate stop time for animal feeding and watering. The continuation of an animal transport after a break is also allowed in Germany if the legally required breaks (Regulation (EC) No 561/2006) were observed. The responsibility of the transporter or transport organization for the animals in the transport process in Germany starts with the loading of the first animal on the vehicle and ends when the last animal has left the vehicle. In Brazil, the responsibility for the animals is with the slaughterhouse. It starts with the loading point of origin to the time of slaughter. This differs from Germany/EU, where the transporter is responsible for the animals from loading to unloading.

It is important to keep in mind the geographical differences between Germany and Brazil. Germany presents a temperate seasonal climate, with mild warm summers (high 20–25 °C) and cold humid winters (low 0–5 °C), while Brazil has a tropical and subtropical climate, with hot summers (high 30–40 °C) and mild winters (10–20 °C). Although Brazil is 24 times larger in area than Germany, travel times from farm to slaughterhouses need not to be longer in Brazil than in Germany or the EU. Most transport of live animals for slaughter in Brazil (and many other South American countries) usually occurs within relatively short distances (300–500 km), with less frequency in long distances (1000–1500 km) or long durations [78]. Most transports of farm animals last between 1 h and 12 h, occasionally reaching up to 60 h long. Such differences have reflected the uneven distribution of paved and unpaved (gravel) road networks, bad weather conditions and intermediate traders, strongly risking animal welfare. Other factors which can negatively influence the welfare of transported animals are poor animal handling on loading, (overstocking) transport and unloading, which is often carried out by uncertified personnel and due to a lack in veterinarian supervision [78]. These may also be reasons for the transport losses in Brazil being estimated as somewhat higher than those in Germany/the EU. Clear legal regulations regarding animal transport—effectively enforced in practice—and proper species-appropriate handling of the animals can help reduce and prevent suffering and losses during transport.

Nevertheless, Brazil and the other Mercosur founding countries, including Uruguay, Paraguay, Argentina and Chile, have been more developed and pay attention to animal welfare, while central and northern countries often still prioritize sociocultural issues over farm animal welfare [79].

It is also worth mentioning that Germany/EU only partially meets its own standards, e.g., live cattle exports to third countries. However, Germany/EU are setting binding minimum standards, while Brazil largely leaves animal welfare to the market and individual states.

Implementation and enforcement show several differences. In Germany, the 431 municipal veterinary offices are responsible for monitoring and enforcing animal welfare. They verify compliance with the Animal Welfare Act and the Animal Welfare Transport Ordinance. In practice, enforcement is carried out through official orders. In Brazil, the monitoring and enforcement of animal welfare for farm animals is primarily the responsibility of the Ministry of Agriculture, Livestock and Food Supply (MAPA), which utilizes a state inspection system with 72 focal points distributed throughout Brazil. In practice, however, compliance is increasingly regulated by private self-monitoring within the meat industry and international standards like WOAH recommendations. For export purposes, the regulations of the importing country are adopted as far as possible.

Effective protection of animals during transport against stress, pain, injury, suffering and death is not merely a matter of animal welfare; it also a matter of educating animal handlers and informing the society about animal-friendly housing and managing. Animal health and welfare generally contribute to the conservation of resources and, ultimately, to the One Health concept, which is based on the premise that the health of humans, animals, the environment and plants within their ecosystems is interlinked. This is also reflected in the 17 Sustainable Development Goals of the United Nations (UN) (UN Doc A/RES/70/1) and forms part of the European Union’s ‘Farm-to-Fork’ strategy [20].

It is very important, however, is to create trust and understanding when comparing two legal systems, which were developed under different geographical, climatic and societal and cultural conditions, and serve the duties in the respective countries. The common aim in Brazil as well as in Germany/EU is to improve the quality of life of our farm animals, particularly during transport, to respect them as fellow creatures and to provide a growing world population with healthy and sufficient food of animal origin. One part of this objective is effective animal welfare regulations, that should be enforced as effectively as possible in practice for the benefit of animals, people and the environment around the world so that all can benefit.

## 6. Conclusions

This comparative review of farm animal transport welfare legislation in Brazil and Germany/EU shows differences and similarities in the number and precision of the regulations. Both countries share the commitment to protect animals from harm, suffering, pain, injuries and death during the whole time of transit. This fundamental mutual understanding of animal welfare is also demonstrated by the fact that the protection of animals is part of their constitutions.

Differences exist in the structure, precision and enforcement of the respective legal frameworks. In Germany/EU, animal transport is regulated through a centralized and detailed legal system, primarily based on Council Regulation (EC) No 1/2005 and the German Animal Welfare Transport Ordinance. These two provisions define transport responsibilities, journey times, rest periods, space allowance, vehicle requirements, driver training, documentation and species-specific conditions.

In Brazil, animal transport is governed by a broad and fragmented set of federal, state- and sector-specific regulations. Although important legal instruments exist, including rules on animal transit documentation, vehicle requirements and specific provisions for certain species or production systems, the overall framework remains less uniform and less detailed than the German/EU system. Transport duration, as an example, varies considerably between states and animal categories. This fragmentation limits legal clarity, harmonized implementation and consistent animal welfare protection across the country.

The comparison also demonstrates that legislation must be understood within the geographical, climatic, infrastructural and socioeconomic contexts of each region. Brazil faces specific challenges related to tropical and subtropical climates, long distances in some production chains, uneven road infrastructure and major regional differences. Germany/EU, by contrast, operates under more harmonized legal conditions, well-developed infrastructure and generally shorter distances in most transport chains and a more established system of official veterinary control, although practical enforcement challenges also remain.

Overall, the findings indicate that animal welfare during transport depends not only on legal standards, but also on practical implementation, professional training, vehicle design, journey planning, enforcement capacity and the commitment of all actors involved. The shared recognition of animal welfare as a relevant legal, ethical, societal and production-related objective provides a strong basis for technical cooperation, mutual understanding and constructive dialog between Brazil and Germany/EU. Such cooperation may be particularly relevant in the context of international trade discussions, including the agricultural chapter of the EU–Mercosur Agreement. Animal welfare standards should be treated as an area for cooperation rather than merely as a trade barrier.

## 7. Recommendations

Brazil should be encouraged to develop a more harmonized national framework for farm animal transport welfare with standards for journey duration, rest periods, feeding and watering intervals, journey breaks, space allowances, ramp design, ventilation, climate protection and emergency procedures. This could be developed along with the adoption of a Federal Animal Welfare Act effectively in all 26 states and the Federal District.

Brazilian legislation should also become more quantitative and species-specific and should use, instead of general terms such as “adequate space”, “proper ventilation” or “appropriate handling”, measurable criteria, e.g., temperature limits and journey duration. Rules on fitness for travel should be expanded and applied consistently to all farm animal species and transport purposes. Training and certification of drivers, handlers and attendants should be strengthened. Vehicle requirements should be further developed, particularly for Brazilian climatic and road conditions.

Documentation (e.g., the Animal Transit Permit) and traceability systems should be linked more directly to animal welfare monitoring including information on animal fitness, loading density, expected journey duration, planned rest stops, vehicle suitability and responsible person. Digital systems could improve transparency and control.

Enforcement should be strengthened through clearer coordination between federal, state and municipal authorities, regular audits, risk-based inspections, roadside checks and slaughterhouse-based welfare monitoring including systematic data collection.

International cooperation should be expanded through the exchange of scientific knowledge, inspection experience, training materials and best practices in vehicle design, logistics and welfare certification. WOAH standards and animal-based welfare indicators could serve as common reference points.

Finally, animal welfare should be integrated more clearly into international trade discussions. It should be understood as a shared goal for sustainability and the One Health objective. A transparent and science-based comparison of welfare standards can strengthen trust, reduce misunderstandings and promote fairer cooperation between livestock-producing regions.

## 8. Limitations of the Study

This study focuses on the core federal Brazilian legal provisions and official guidance relevant to the road transport of farm animals. It does not provide a complete survey of all state-level or municipal rules, administrative practices, or sector-specific implementation measures because Brazilian animal transport regulation is partly fragmented across federal, state, and local levels, and amendments may not always be centrally consolidated or easily accessible. Therefore, it cannot be guaranteed that every recent amendment to state-level regulation has been fully captured. The study is also limited by the availability of official sources, language accessibility, and the extent to which enforcement practice, inspection data, and unpublished administrative guidance are publicly available. Accordingly, the findings should be understood as a structured comparative overview rather than as an exhaustive legal opinion on all applicable Brazilian transport requirements.

## Figures and Tables

**Table 1:** Comparison of German and Brazilian regulations on animal transport vehicles.

Germany/EU	Brazil
Council Regulation (EC) 1/2005	CONTRAN 791/2020 [31]
Annex I Chapter II. 1.1	Art. 3
(a) avoid injury and suffering and ensure the safety of the animals	I—constructed or adapted and maintained to avoid unnecessary suffering, injury and agitation, ensuring life and welfare
(b) protect from adverse weather conditions, extreme temperatures and climatic fluctuations	VIII—protective devices to minimize the effects of extreme temperatures must be present
(d) prevent the animals escaping or falling out and be able to withstand the stresses of movements	XIII—sides and a roof that protect against escape, falls and the outside protrusion of body parts must be present
(e) ensure that air quality and quantity appropriate to the species transported can be maintained	VII—the air circulation inside the vehicle must guarantee the ventilation necessary for the welfare of the animals
(g) the floor surface is non-slip	XI—non-slip floor surface to prevent slipping and falling outside the restraint containers
(h) the floor surface is designed such that the leakage of feces or urine is kept to a minimum	X—devices to prevent the discharge of excrement while driving on public roads must be present

**Table 2:** Space requirements for cattle transport by land in Brazil and Germany/EU.

Germany (Council Regulation 1/2005) [19]		Brazil (MAPA 46/2018) [35]	
Cattle Weight (kg)	Space (m^2^)	Cattle Weight (kg)	Space (m^2^)
110	0.4–0.7	100	0.31
200	0.7–0.95	200	0.53
325	0.95–1.3	350	0.98
550	1.3–1.6	550	1.34
≥700	≥1.6	≥600	1.63

**Table 3:** Journey limits for farm animal road transport in Brazilian states.

Maximum Journey Time. Obligation to Take a Break (Hour)	Applies in Following Federal States	Law. Regulation	Reference
4	Amazonas	6.670/2023	[63]
6	São Paulo	11.977/2005	[64]
6	Maranhão	10.169/2014	[65]
6	Sergipe	8.366/2017	[66]
6	Rio Grande do Norte	10.831/2021	[67]
6	Ceará	17.729/2021	[51]
6	Piauí	8.364/2024	[50]
12	Brasília	4.060/2007	[68]
Mandatory break after 6 h	Rio de Janeiro	RJ 11.096/2026	[69]
Mandatory break after 12 h	Paraná	14.037/2003	[70]
Mandatory break after 12 h	Santa Catarina	12.854/2003	[71]
Mandatory break after 12 h	Espírito Santo	8.060/2005	[72]
Mandatory break after 12 h	Pernambuco	15.226/2014	[73]
Mandatory break after 12 h	Tocantins	3.530/2019	[74]
Mandatory break after 12 h	Rio Grande do Sul	15.363/2019	[75]
12	Nationwide organic farms	MAPA 46/2011	[34]

## Data Availability

No new data were created or analyzed in this study. Data sharing is not applicable to this article.
